# GraphoLearn India: The Effectiveness of a Computer-Assisted Reading Intervention in Supporting Struggling Readers of English

**DOI:** 10.3389/fpsyg.2018.01045

**Published:** 2018-06-26

**Authors:** Priyanka Patel, Minna Torppa, Mikko Aro, Ulla Richardson, Heikki Lyytinen

**Affiliations:** ^1^Department of Education, University of Jyväskylä, Jyväskylä, Finland; ^2^Department of Teacher Education, University of Jyväskylä, Jyväskylä, Finland; ^3^Centre for Applied Language Studies, University of Jyväskylä, Jyväskylä, Finland; ^4^Department of Psychology, University of Jyväskylä, Jyväskylä, Finland; ^5^Niilo Mäki Institute, Jyväskylä, Finland

**Keywords:** GraphoLearn, reading intervention, computer-assisted learning, phonics, grapheme-phoneme correspondence, English language learners, India

## Abstract

India, a country with a population of more than 1.3 billion individuals, houses the world’s second largest educational system. Despite this, 100 of millions of individuals in India are still illiterate. As English medium education sweeps the country, many are forced to learn in a language which is foreign to them. Those living in poverty further struggle to learn English as it tends to be a language which they have no prior exposure to and no support at home for. Low-quality schools and poor instructional methods further exacerbate the problem. Without access to quality education, these individuals continue to struggle and are ultimately never given the chance to break the cycle of poverty. The aim of this study was to determine whether GraphoLearn, a computer-assisted reading tool, could be used to support the English reading skills of struggling readers in India. Participants were 7-year-old, grade 3 students (*N* = 30), who were attending an English-medium public school in Ahmedabad, India. English was not a native language for any of the students and all were reading at a level below that of Grade 1 despite having attended school for 2 years. Half of the students played GraphoLearn (*n* = 16) while the other half played a control math game (*n* = 14) for 20–30 min a day, over a period of 8 weeks. GraphoLearn led to significant improvements in children’s letter-sound knowledge, a critical factor in early reading development. Overall, the study opens doors for GraphoLearn as a potential intervention to support struggling readers of English in India, including those who are learning a non-native language and coming from at-risk backgrounds.

## Introduction

Despite international moves and agreements to improve literacy around the world, many developing countries are still struggling with high rates of illiteracy. India, a country with a population of 1.3 billion individuals, only has a literacy rate of 72% among those 15 years and older (UNESCO, 2015). In a country developing as quickly as India, an illiteracy rate which leaves 100 of millions as illiterates is highly concerning as it puts many individuals at risk of never being able to reach opportunities and act as contributing members of society. With 17 official languages (as recognized by the United Nations) and more than 700 dialects (Mitra et al., 2003; Dixon et al., 2011), and with 21% of the population, or 269 million people, living below the poverty line (The World Bank, 2011), solving India’s literacy crisis is an extremely large task.

Education plays a major role in literacy and, therefore, some believe that one strategy to start combatting the problem may be to look at countries with successful education systems and borrow interventions that can be implemented elsewhere (Ojanen et al., 2015). Children in India, especially those living in poverty, face many problems in education. Slum and other low-income children are forced to attend low quality schools, which are under-resourced and use poor teaching methods (Cheney et al., 2005; Kingdon, 2007). With a country-wide push towards English medium education, these students are studying in a language which they may have no prior exposure to and no support at home for. Due to factors such as these, many children struggle to learn English and attain a quality education. In turn, many of these children will never have the option of higher education, and once again, they will find themselves stuck in the cycle of poverty. According to The World Bank (2012) 45% of the poor are illiterate as compared to 26% of the non-poor.

The purpose of this study was to determine whether GraphoLearn, a computer-assisted tool for reading instruction, originally created for struggling readers of Finnish, could be used to support struggling readers of English in India. The major focus is on slum children attending government-aided public schools in Ahmedabad, India, who are non-native speakers of English, and at high risk of never achieving fluent English literacy.

### English in India

English as a language was originally brought to India by the British who arrived in the 1600s and established trade posts through the East India company (Mehrotra, 1998). English was used throughout the British rule between traders and merchants, as well as by Christian missionaries (Mehrotra, 1998). During this time, English was viewed as a language of the elite, a view that has been upheld even post Indian independence in 1947 (Mishra and Stainthorp, 2007). Being that India is a highly multicultural country, English has been maintained, and acts as a common bridging language across states (Mitra et al., 2003). British rule brought with it a tradition of English medium education to India (Annamalai, 2004) which was maintained as there was no other language throughout the country which would be accepted by the linguistic minorities (Mishra and Stainthorp, 2007).

In present day India, it is common for individuals to use a variety of languages in everyday life (Mishra and Stainthorp, 2007). It may even be that one language is used in the workplace or school, while another language is used in speaking to peers, and then the mother tongue is used in speaking to family and other relatives. Today, English is the only language that is taught in all states and in the most number of schools across the country (Annamalai, 2004). Individuals who speak English are coveted by employers (Mitra et al., 2003; Annamalai, 2004) and it has become a very important language, particularly in higher education (Mehrotra, 1998; Annamalai, 2004; Cheney et al., 2005), with the majority of high level institutions only providing instruction in English. As a result, English has the ability to influence the standard of living in India; with those having better English skills getting better job opportunities, and in turn better pay (Mehrotra, 1998; Mitra et al., 2003). As parents realize the opportunity that comes with learning English, many are actively choosing to enroll their children in English medium schools. This is true even for parents from slum areas who have started accepting that the ability to read, write, and speak in English will increase opportunity for their children (Mehrotra, 1998; Mitra et al., 2003; Dixon et al., 2011). Currently, there are 90 million children across various socioeconomic statuses that are becoming literate in English (Kaila and Reese, 2009).

However, children growing up in slum communities are at a large disadvantage when it comes to learning the English language (Annamalai, 2004). In English medium schools, English is the primary language of instruction, meaning that all subjects are taught in English, with regional and other languages taught as second and/or third languages. Slum children often have no exposure to English prior to entering school, as parents typically cannot speak or communicate in English. It is also likely that these parents are illiterate in their mother tongue as well (Dixon et al., 2011), meaning that their children will have no exposure to literacy in any language prior to school entry. According to Nag (2013) children who miss such supports, such as having a print rich environment with access to reading material or an adult to read to them, tend to develop profiles which are similar to those with dyslexia or other reading difficulties. Thus, children are at high risk even before they enter the school.

Parents from the lower levels of society, typically have two choices in terms of schools for their children; government -aided public schools or low-income, unaided private schools (Cheney et al., 2005). Due to the high demand for English, there has been a “mushrooming” of low-cost private schools (Tooley and Dixon, 2005), and now English is also taught as a primary language in public government schools. In most of these public and private schools, teaching quality is low and children are forced to rote learn a language they do not fully understand (Annamalai, 2004; Dixon et al., 2011). On the contrary, there are many private schools across the country which follow international board curriculum and provide high quality English education. However, these schools charge high fees making them inaccessible to the low-income population (Cheney et al., 2005).

According to the latest Annual Status of Education Report (ASER), 95.9% of children ages 6–14 are enrolled in school across India (2016). Although school enrollment is high, learning achievements of these enrolled children are consistently low (Kingdon, 2007). Across all languages, only 47.8% of children in Grade 5 are able to read a Grade 2 level text (ASER, 2016). When looking at English, of all surveyed children in Grade 3, only 19.3% could read simple words such as “day” or “sit” (ASER, 2016). Although the ASER report only surveys children in rural India, data from the National Achievement Survey (NAS) shows that the situation in urban India is not strikingly different. The NAS for Grade 3 students has three measures on the language assessment; listening comprehension, word recognition, and reading comprehension. Across the nation, the average score was 257 out of a total 500, leaving approximately 50% of Grade 3 students unable to perform at grade level (NCERT, 2014).

### Grapheme-Phoneme Correspondences and Early Reading Acquisition

Learning to read in any language requires understanding the links between the spoken language and its written form. More specifically, those who are learning to read must understand the grapheme-phoneme correspondences (GPC’s) that occur within a particular language. It has been well established that knowledge of grapheme-phoneme correspondences directly impacts fluent reading (e.g., Ehri, 2005) and such knowledge is necessary for further development of reading skills.

However, the ease of reading acquisition is greatly determined by the orthographic depth of a language. Many researchers agree that reading acquisition in English, is much more complicated than reading acquisition in many other languages, due to its deep orthography (see Seymour et al., 2003). The grapheme-phoneme correspondences in English are more complex and context-dependent and therefore, there is still some disagreement on how early reading instruction in English should proceed. Some argue that English, and other opaque orthographies, might be more effectively introduced through larger units, also known as rime units, rather than at the level of single graphemes and phonemes (Goswami, 1986, 1988), as they tend to be more consistent. It is believed that English-speaking children may benefit more if focus is put towards teaching these larger rime units and can then use rime analogies from words that they already know to read unfamiliar words as well (Goswami and Bryant, 1990).

However, when compared to instruction based on small units, some studies have failed to find any significant differences when comparing instruction based on grapheme-phoneme correspondence as compared to onset rime (e.g., Haskell et al., 1992; Levy and Lysynchuk, 1997). A study conducted by Christensen and Bowey (2005) compared children participating in two explicit, decoding programs, one which was based on orthographic rimes and a second which was based on grapheme-phoneme correspondences. The study also involved a control group which received implicit phonics instruction. Not surprisingly, it was found that both of the explicit instruction groups outperformed the implicit control group in reading and spelling. Interestingly, the study also showed differences between the orthographic rime group and the grapheme-phoneme correspondences group, with the grapheme-phoneme correspondences group performing better at reading and spelling unfamiliar words. The role of grapheme-phoneme correspondences in reading development have also been established amongst children who are non-native speakers of English. Researchers in Canada compared children who were either native speakers of English or native speakers of Punjabi, all of whom were attending school in English. They found that both groups of students were reliant on grapheme-phoneme correspondences when they were presented with unfamiliar words. Similarly, for both groups, errors in reading were due to the inability to apply grapheme-phoneme correspondences to unfamiliar words (Chiappe and Siegel, 1999) with poor readers being less skilled at this application.

Nevertheless, there tends to be consensus that early reading instruction through phonics (individual phonemes or onset-rime) should follow a systematic approach in which children are taught to connect spoken language segments to their corresponding written forms (Wyse and Goswami, 2008; Kyle et al., 2013). Automatization of this phonetic knowledge of a language plays a critical role in early reading development and later reading skill (Ehri, 1998; Juel and Minden-Cupp, 2000).

### Reading Instruction: From Rote Memorization to Systematic Phonics

Children studying English in India, particularly those in low-income schools, are taught English in a rote manner (Annamalai, 2004; Dixon et al., 2011). Students learn the names of letters, rather than sounds, and are then expected to learn “common” words as a whole in which students essentially learn to recognize words through sight. Like words, sentences are also learned through a method of rote memorization in which someone points to the words written on the board, which are then chanted by the rest of the class (Dixon et al., 2011). Through such rote learning methods, children are unable to blend or decode unfamiliar words and are therefore, only able to “read” words which are familiar to them, but that too often with limited comprehension. The NAS uses reading comprehension as the primary measure of language knowledge of Grade 5 students across India. In 2015, it was found that nationally Grade 5 students only scored an average of 48.2% (out of a total of 100%) on the reading comprehension assessment (NCERT, 2015). Thus providing evidence against such rote methods of reading instruction to teach English in India.

One of the most popular methods of early reading instruction in English-speaking countries has been through systematic phonics. The phonics approach involves explicitly instructing readers on the linkages that exist between letters and their corresponding sounds, and how that is then used to read words. Synthetic phonics approaches, in which children learn small units of language (graphemes and phonemes) are believed to be the most logical way to support early reading development (e.g., Seymour and Duncan, 1997; Hulme et al., 2002). Major correspondences are taught, as well as vowel sounds, digraphs, blends, onsets, and rimes (Ehri et al., 2001). There is ample support for systematic, synthetic phonics programs among native speakers of English (e.g., Ehri et al., 2001; Johnston and Watson, 2005). Fortunately, there is also strong evidence in favor of synthetic phonics programs for children learning English as a second language. A study by Stuart (1999) looked at reading instruction for 5-year-old children through a synthetic phonics program, Jolly Phonics, versus a more holistic program which placed no explicit importance on phonics. Majority of the sample (*N* = 96 out of 112) were children who were learning English as a second language. Results showed a significant positive effect of the Jolly Phonics intervention on the children’s reading and writing development which persisted even a year after the initial intervention. Based on these results, researchers concluded that early structured, rapid, and focused teaching of phonetic manipulation actively supports development of this knowledge, even for children who are non-native speakers of the language (Stuart, 1999). A follow up study by Stuart also showed that even if children have not been taught using phonics at the start of school, they can catch up through structured and intensive phonics training (Stuart, 2004).

Such findings of the effectiveness of phonics teaching among second language learners is important for the Indian context as children in India are predominantly bilingual (and in some cases even multilingual), which creates a unique educational situation. Most children are exposed to their mother tongue prior to entering school, upon which they may begin to study in a language which they have no previous exposure. If the mother and father happen to speak different languages, then they may already encounter two different languages before starting formal schooling (Mishra and Stainthorp, 2007).

Synthetic phonics approaches have made their way to developing countries more recently; India being one such country of study. Dixon and colleagues tested the Jolly Phonics intervention with children attending English-medium, low-income private schools in Hyderabad, India. There was an experimental group which received the intervention for an hour per day for 6 months by the teacher, and a control group which received the traditional English instruction, typically involving rote-learning and whole word recognition. Results showed a statistically significant difference between the experimental and control groups, with the experimental group performing better on tasks of reading, spelling, and sounding out letters and words (Dixon et al., 2011). Effect sizes (*d*) were particularly strong for tasks assessing sound value of letters (16.18), blending (1.20), sentence dictation (1.01), and spelling (.86). Findings such as these strongly support the idea that phonics interventions could be successful to improve emergent English literacy in India.

### Why Technology?

As it can be seen, there are a number of factors working against slum community children in India, when it comes to learning to read in English. Coming from homes, where parents may also be illiterate, children are suddenly forced to learn in a language which they may have no prior exposure to. Mother tongue instruction also may not be seen as an ideal option in a place like India, where English is given such high importance and has the potential to open many more doors. However, the rote methods teachers are currently using are clearly not helping students to achieve. Thus, the children are put in a situation where, although they are attending English medium schools, they may never acquire sufficient English literacy. The few studies which have been done using synthetic phonics instruction to teach English in India have produced promising results (Dixon et al., 2011). However, due to the numerous demands faced by teachers in India, as well as a potential lack of skill, changing instructional methods may seem intimidating for many. Technology, on the other hand, has the potential to help teachers overcome some of these barriers, and in turn allow them to provide the high-quality literacy instruction that all children deserve.

India has always been a strong player in the IT industry (Mitra et al., 2003; Kingdon, 2007). The Indian Market Research Bureau along with the Manufacturers’ Association for Information Technology (MAIT-IMRB) has reported the tablet market in India to be growing at a rate of 73% (as cited in Central Square Foundation, 2015). Smartphone use is also becoming widespread as more and more low cost models come on the line (Central Square Foundation, 2015). As a result, the Indian government has also been actively working to integrate technology into the educational space through various initiatives. One such initiative is the “ICT@Schools” scheme. According to the Ministry of Human Resource Development, the government has spent 2585 crore Indian rupees (approximately 38 million USD), to install technological infrastructure in about 86,000 schools across the country (as cited in Central Square Foundation, 2015).

Researchers have found that not only is technology-led instruction benefiting children’s learning (Banerjee et al., 2007), it is also cost effective and time effective (Muralidharan et al., 2017). Insights from studies across the educational technology sector in India have shown the benefits of, and continuing need for, technology that allows for differentiated instruction through personalized learning (Central Square Foundation, 2015). Though technology is greatly influencing modern educational spaces, there has been criticism against solely using technology as an intervention. A meta-analysis comparing technology use for direct versus support instruction resulted in a slightly greater effect for support instruction (see Tamim et al., 2011). Supporting results have been found when technology as a teacher compliment versus a teacher substitute was studied in the context of India. Linden (2008) found that students who received a math intervention as a substitute to teacher delivered curriculum performing significantly worse than students who received the intervention as a compliment to teacher instruction. Similarly, a study comparing the effects of a computer-based intervention to teacher implemented activities found that different students benefited from different interventions, with the lower performing students benefiting more from the teacher implemented activities and the higher performing students benefiting more from the computer-based intervention (He et al., 2008).

### The GraphoLearn Method

GraphoLearn,^1^ previously known as GraphoGame, is a theoretically driven computer-assisted tool for early reading that provides training on the connections between spoken and written language by explicitly instructing on grapheme-phoneme correspondences. The structure of the game is based on a theory of teaching small units, or 1–2 phonemes first, as this phonetic knowledge has been shown to be a strong predictor of later reading skill (e.g., Seymour and Duncan, 1997; Hulme et al., 2002). It was originally devised for readers of a transparent orthography, Finnish, based on longitudinal data that was collected through the Jyväskylä Longitudinal Study of Dyslexia (Lyytinen et al., 2007, 2009; Richardson and Lyytinen, 2014). The Finnish version of GraphoLearn has been adapted to other languages around the world, English being one, and results have been promising in many countries across various languages (e.g., Saine et al., 2011; Kyle et al., 2013; Ojanen et al., 2015; Ruiz et al., 2017). To date, there has been no study which has used GraphoLearn to support non-native speakers of English.

There are two GraphoLearn English versions GraphoLearn English-Rime and GraphoLearn English-Phoneme. Prior to the current study, there has only been one published study done investigating GraphoLearn English. Kyle et al. (2013) tested the efficacy of the two versions of GraphoLearn English as a supplementary tool for students who were native English speakers in the United Kingdom. Results showed significant improvements in basic reading skills of the intervention group as compared to the controls for both game versions, but were unable to conclude that one version was more effective than the other. In the present study, GraphoLearn English-Rime was utilized. It incorporates the idea of teaching slightly larger rime units in addition to single grapheme-phoneme correspondences due to the orthographic complexity of English as a language (e.g., Goswami, 1986, 1988). In both game versions players are first introduced to single grapheme-phoneme correspondences. However, rather than introducing them all at once, in GraphoLearn English-Rime, grapheme-phoneme correspondences are introduced in sets of about 7–8 items. These individual letters are then combined to form rime units, and finally whole words. Later in the game, players are also shown whole words in which they must isolate or blend various grapheme-phoneme correspondences or rime units. Presentation of the grapheme-phoneme correspondences proceeds from the most frequent and consistent to the more infrequent and least consistent (Kyle et al., 2013). Kyle et al. (2013) reported that for the game version used in this study, effect size was large for BAS spelling (0.66) and TOWRE non-word reading (1.43) and medium for BAS reading (0.66) and TOWRE sight word reading (0.53) (Kyle et al., 2013).

### The Present Study

The study reported here examined the efficacy of GraphoLearn, a computer-assisted reading tool, in improving basic reading skills of English by supporting the development of grapheme-phoneme knowledge, reading, and spelling ability of slum children in India. GraphoLearn was provided as a supplement to teacher instruction to third grade students in an English medium, government-aided public school in Ahmedabad, India. The school was approached based on information retrieved from the class teacher which showed the children as having very low literacy levels. We chose Grade 3 in order to assume that the children had at least 2 prior years of spoken English exposure (starting from Grade 1). Based on previous studies using synthetic phonics (Stuart, 1999; Stuart, 2004; Dixon et al., 2011) and based on previous GraphoLearn studies (Kyle et al., 2013), we expected to see improvements in student performance.

## Materials and Methods

### Ethics Statement

Permission to run the study was taken from the Ahmedabad Municipal Corporation School Board, along with the principal and the class teacher. Parents of the children (both pilot and full study) provided written informed consent prior to the start of the intervention. The study was carried out in accordance with guidelines as given by the University of Jyväskylä Ethics Committee. An ethics approval was not required as per the University of Jyväskylä Ethics Committee guidelines and national regulations. However, a statement from the Ethics Committee can be provided upon request.

### Pilot

Prior to the start of the full study, a pilot was conducted including 16 children from a second government-aided public school. These students were also in Grade 3 and had similar demographics as the children who participated in the full study. The pilot phase was run for 3 weeks and the primary purpose of the pilot phase was to experience the type of difficulties which may arise in the full study in a hope to circumvent such difficulties later. After the pilot period, there were some changes that were made prior to the start of the full study. The math game was changed for the controls as the original game which was selected was not long enough for students to play throughout the entire study period. Another change was to the paper-pencil tasks. It was originally planned to conduct a standardized phoneme deletion task as used by Kyle and colleagues (Kyle et al., 2013). However, when attempted with the children during the pilot, it was obvious that most children did not understand the task. Therefore, the standardized phoneme-deletion task was not included in the full study.

### Participants

Thirty-one third graders, ages 7–8 participated in the study. Data provided by the teacher showed that the children, on average, were performing drastically below grade level in literacy. Due to the lack of specialists in the school, it was unknown if any children had additional special needs in learning, but no students had any formal diagnoses of such problems. All of the participating students were consented, at the end of second grade before they left for summer holidays to ensure that the study could begin as soon as possible once they returned. Parents were invited to the school and taken through the consent form as many were illiterate in English. In total, 43 parents provided written informed consent, however, only 31 children ended up participating in the study as some children dropped out of the school prior to the start of the study while other children had extremely irregular attendance or joined the school after the start of the study and therefore could not be included.

Students were randomly allocated to either the experimental group which played GraphoLearn (*n* = 16) or the control group which played a math game (*n* = 15). Groups were primarily matched based on age and gender, but basic reading skills, such as letter-sound knowledge, were also considered based on the information provided by the teacher. All students came from low-income homes, with a majority living below poverty line, and all students were learning English as a second or third language, with no exposure at home to English. All the children, except for one, had been enrolled in the school from Grade 1 and they had all been in the same classroom with the same teacher in both Grades 1 and 2. At the end of the study, there were three students who were unable to participate in all or some parts of the post-test due to illness. One student’s data from the control group has been removed because they did not participate in any of the post testing. The other two students’ data, both of whom were in the GraphoLearn group, was not removed because one participated in the GraphoLearn post-tests and the other participated in the paper-pencil post-tests. Significance values and effect sizes were not affected by eliminating these students’ data, and therefore their data has been retained. Final group sizes at post-test were *n* = 16 for the GraphoLearn group and *n* = 14 for the control group. As a reward for the participation and cooperation of the class teacher and students involved, a set of 20 English story books were donated to the classroom at the end of the intervention period.

### Procedure

Both groups of children played their respective games (GraphoLearn versus math) for 20–30 min per day, 6 days a week, over a period of 8 weeks. The children played the game on an individual tablet with headphones. All play was done during the regular school day where children were pulled out of their classroom in batches of 12 and then taken to a separate room where the tablets were set up for them. The researcher was present during all play sessions with the students.

### GraphoLearn

**FIGURE 1:**
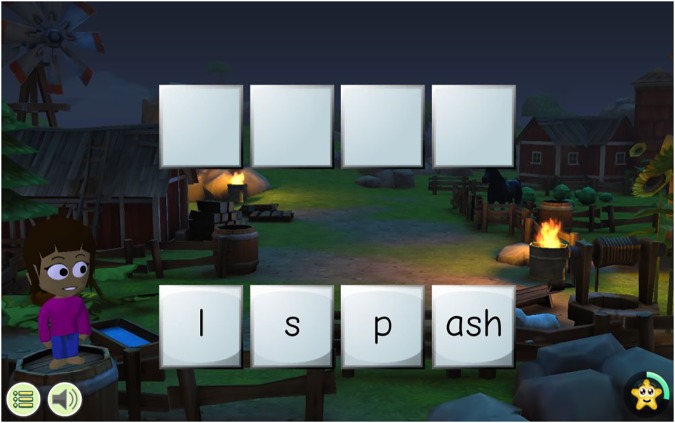
An example of the screen during a word formation task in stream six.

GraphoLearn provides adaptive practice in which players see a set of letters or letter strings and hear a corresponding speech sound. Players are expected to select the correct written unit from the 4 to 7 options that correspond to the sound they hear from the headphones. GraphoLearn requires players to create an individual avatar after which they are taken through a series of streams which are divided into levels GraphoLearn English-Rime has a total of 25 streams. Each stream contains anywhere from 5 to 9 levels. The first seven streams start with a level with introduces players to a small set of individual grapheme-phoneme correspondences (7–8 items), some of which are new and others which are review from previous streams. Once these are introduced, they are then combined to form larger rime units. These larger units are then presented in the context of words. Further in the game, players are introduced to more complex grapheme-phoneme correspondences (e.g., blends and digraphs) and sounds which have multiple possible spellings. After every four streams, there is an assessment stream in which players are assessed on letter-sounds, rime units, and word recognition. Throughout the game, players are presented with auditory targets which they then must match with the correct visual target out of items presented on the screen. The streams are ordered according to difficulty, starting from the easiest and progressing to the more difficult connections present between spoken and written English. To support spelling skills, word formation levels are present in 15 streams. Players are presented with blocks on the screen containing either individual letters or onset and rime patterns which they then have to drag into boxes in the correct order to spell a target word (see **Figure 1**). In order to further support the development of phonological awareness, there are rhyming tasks present in 11 of the streams requiring players to select the target that rhymes with the auditory target they are presented with. In all the levels, if players choose incorrectly, they are provided with automatic feedback, allowing them to correct themselves. Players must score above 80% on each level within a stream in order to move on to the next stream. To further build motivation, players are rewarded within each level with stars and coins which they can trade in to purchase things for their avatar. Data from the game is automatically saved to an external server when players exit the game so long as the device has an active internet connection (For a detailed description of GraphoLearn English see Kyle et al., 2013).

### Math Game

The math game played by the control group was a Grade 3 level game called “Math for Kids” selected from the Google Play store. It provided students with basic operations problems (addition, subtraction, and multiplication) and students were required to select the correct answer out of four targets provided. Students could select out of three degrees of difficulty (easy, medium, and hard) and their progress in the game was saved meaning they could continue every session where they last left off. The math game was similar to GraphoLearn in that within each level there were multiple sublevels. The game rewarded children with stars and children were instructed to move on to the next level only after collecting at least two stars. The game provided no visual or auditory English input other than at the beginning when children had to select their level. The main purpose of the math game was to ensure that both groups of children spent equivalent amounts of time in the classroom versus outside of the classroom using the technology. As it can be seen in **Table 1**, there were no significant differences in the number of days played or playing times between the two groups.

**Table 1:** Group characteristics.

Characteristic	GraphoLearn	Control	
*n* (Pre-Test)	16	14	–
*n* (Post-Test)	15	14	–
**Gender**		
Male	8	7	–
Female	8	7	–
Age (months)	91.94 (0.63)	91.00 (0.84)	*t*(28) = 0.91
Playing time (min)	470.7 (40.8)	457.3 (68.0)	*t*(20.7) = 0.64
Playing days	21.3 (1.7)	20.8 (3.1)	*t*(19.5) = 0.50

### Measures and Assessment Procedure

Students were assessed at pre and post intervention using three tasks in the GraphoLearn software and four paper-pencil tasks. The in-game assessment included the following tasks: letter-sound knowledge, rime unit recognition, and whole word recognition. The standardized paper-pencil tasks included the following tasks: the Single Word Reading subtest from the British Ability Scale (BAS II; Elliot et al., 1996), and the Test of Word Reading Efficiency (TOWRE; Torgesen et al., 1999) which included sight word reading and non-word reading. Students also completed a modified version of the spelling subtest from the BAS II. Students were pre-tested and post-tested by the researcher in the days preceding and the days following the intervention period. Students were pulled out of the class and completed the BAS II reading, TOWRE sight words, and TOWRE non-words tasks one-on-one with the researcher. The BAS II spelling assessment was given as a whole class dictation and the GraphoLearn in-game assessments were given to students in groups of 12 on the tablets. Both the GraphoLearn and control groups were given basic instructions on how the GraphoLearn assessments work prior to the start of the assessment tasks, and all students were instructed to inform the researcher once they finished an assessment task, and prior to starting the next assessment task. Through this, it was ensured that children were not playing levels which they should not be and all three assessment tasks were only being played once at pre-test and once at post-test.

#### In-Game Assessments

**FIGURE 2:**
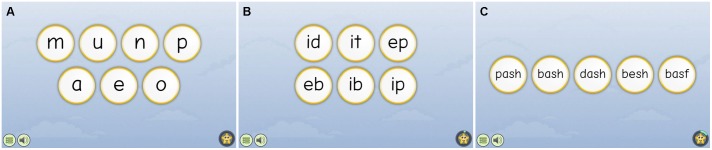
Example screens from the GraphoLearn in-game assessments **(A)** is from the letter sounds task, **(B)** is from the rime units task, and **(C)** is from the word formation task.

All students completed three in-game assessments in GraphoLearn. The letter-sound task required children to pick the correct letter, out of the options, that corresponded with the sound which was presented to them (see **Figure 2A**). The rime unit task required children to pick the correct 2–3 letter string that corresponded to the pronunciation presented to them (see **Figure 2B**). Finally, the word-recognition test required children to pick the correct word to that which was presented to them (see **Figure 2C**). In all three tasks, players were presented with an auditory target which they were required to match with a visual target, just as in the rest of the game. In total, the letter sounds task contained 24 trials, the rime units task contained 24 trials, and the word recognition task contained 47 trials. The game would discontinue for the rime units task and the word recognition task if players chose incorrectly more than 50% of the time. The average number of trials played within all three tasks are given in **Table 2**. Both the experimental and control groups completed the assessment level prior to and at the end of the intervention period.

**Table 2:** Average number of trials completed within the in-game assessments at pre and post-test.

	Pre-test	Post-test
Letter-sounds	24	24
Rime units	4.97	8.10
Word recognition	6.87	8.71

#### Paper-Pencil Assessments: Reading

All students in the study completed the Single Word Reading subtest from the British Ability Scale II (BAS II; Elliot et al., 1996) which measures single-word reading accuracy. The test was administered according to the manual and required children to read single-words of increasing difficulty which are listed in groups of 10. The test is discontinued after children miss eight or more words within one group. Internal reliability of the BAS II word reading task has been reported to be 0.98 and test-retest reliability has been reported to be 0.97 as per test review (Thomson, 1997). Students also completed the Test of Word Reading Efficiency (TOWRE; Torgesen et al., 1999). The TOWRE requires students to accurately read aloud a list of sight words and non-words for 45 s. Practice words were given for each section. Internal reliability ranges from 0.86 to 0.98, and test-retest reliability has been reported to be between 0.82 and 0.97 for both tasks, as per test review (Tanna, 2009). It is important to note that these assessments are not standardized for Indian children and therefore only raw scores are provided.

#### Paper-Pencil Assessments: Spelling

All students also completed a spelling subtest which was taken from the British Ability Scale II (Elliot et al., 1996). The task contained a mixture of verbs, nouns, and adjectives, some of which can be spelled phonetically. The dictation test was not carried out according to the instructions suggesting different starting points based on age. Rather, the first 30 words out of the list were dictated to all students with the accompanying sentence. The word and an accompanying sentence were said a maximum of three times and students were expected to write down the word. The score was the number of correctly spelled items out of 30.

### Fidelity to the Program

Fidelity to the GraphoLearn intervention was controlled by the detailed game logs sent to the GraphoLearn server. These logs include the number of days played and seconds spent playing. The first and last play day were also recorded. For the control group, days and time (in minutes) were recorded manually by the researcher. In addition, the primary researcher was present through all play sessions to ensure that the children were engaged in playing the respective games.

## Results

Prior to analyses, the distributions of all measures were assessed for normality. The BAS II reading measure at pre-test had two scores which were outliers and caused a right-skewed distribution. The TOWRE non-words measure at pre-test had one score which was an outlier and caused a right-skewed distribution. These scores were winzorized (replaced with a value that was closer to the distribution while retaining the order of values) to meet the assumption of normality. The remaining measures (GraphoLearn letter-sounds, GraphoLearn rime units, and GraphoLearn word recognition, TOWRE sight words, spelling) all produced a normal distribution at both time points.

### Pre-test and Post-test Group Comparisons

The pre-test and post-test means and standard deviations in the two study groups, as well as group comparison results, are reported in **Table 3** for the GraphoLearn tasks and **Table 4** for the paper-pencil tasks.

**Table 3:** Descriptive statistics and group comparisons on GraphoLearn tasks.

Measure	Assessment	GraphoLearn *M (SD)*	Control *M (SD)*	*t*	Group effect	Time effect	Interaction effect
Letter-sounds	Pre-Test	33.3% (11.2)	36.3% (8.7)	*t*(28) = -0.81	*F*(1,27) = 12.95^∗∗∗^	*F*(1,27) = 25.91^∗∗∗^	*F*(1,27) = 44.87^∗∗∗^
	Post-Test	63.9% (18.0)	32.1% (10.6)	*t*(27) = 5.73^∗∗∗^			
Rime units	Pre-Test	16.6% (16.7)	13.6% (15.6)	*t*(28) = 0.50	*F*(1,27) = 3.09	*F*(1,27) = 18.24^∗∗∗^	*F*(1,27) = 3.13
	Post-Test	39.4% (20.5)	23.2% (17.0)	*t(*27) = 2.31^∗^			
Word recognition	Pre-Test	30.7% (16.3)	29.2% (19.8)	*t*(28) = 0.23	*F*(1,27) = 1.03	*F*(1,27) = 25.13^∗∗∗^	*F*(1,27) = 2.68
	Post-Test	49.0% (12.1)	39.1% (13.5)	*t*(27) = 2.07^∗^			

*^∗^p ≤ 0.05, ^∗∗^p ≤ 0.01, ^∗∗∗^p ≤ 0.001.*

**Table 4:** Descriptive statistics and group comparisons on paper-pencil tasks.

Measure	Assessment	GraphoLearn *M* (*SD*)	Control *M* (*SD*)	*t*	Group effect	Time effect	Interaction effect
BAS II reading	Pre-Test	15.9 (11.5)	14.4 (12.0)	*t*(28) = 0.72	*F*(1,27) = 0.02	*F*(1,27) = 12.39^∗∗^	*F*(1,27) = 0.72
	Post-Test	19.7 (13.7)	20.1 (18.6)	*t*(27) = -0.07			
TOWRE sight words	Pre-Test	15.6 (9.2)	18.3 (13.7)	*t*(28) = -0.63	*F*(1,27) = 0.15	*F*(1,27) = 10.98^∗∗^	*F*(1,27) = 0.67
	Post-Test	19.5 (12.8)	20.5 (13.2)	*t*(27) = -0.22			
TOWRE non-words	Pre-Test	6.5 (4.2)	7.6 (4.9)	*t*(28) = 0.53	*F*(1,27) = 0.02	*F*(1,27) = 7.86^∗∗^	*F*(1,27) = 1.23
	Post-Test	9.3 (6.3)	8.8 (6.4)	*t*(27) = 0.23			
Spelling	Pre-Test	10.1 (8.5)	12.2 (8.9)	*t*(28) = -0.66	*F*(1,27) = 0.09	*F*(1,27) = 11.95^∗∗^	*F*(1,27) = 3.67
	Post-Test	13.7 (8.1)	13.3 (8.6)	*t*(27) = 0.12			

*^∗^p ≤ 0.05, ^∗∗^p ≤ 0.01, ^∗∗∗^p ≤ 0.001.*

First, an independent samples *t*-test was conducted to examine if there were group differences at pre-test or post-test. Due to the small sample size, group differences were also analyzed using non-parametric measures (Mann–Whitney *U*) but as the results did not differ from those given by the *t*-test, and therefore, the *t*-test results are reported. Effect sizes and their confidence intervals at pre-test were also calculated for all measures using Cohen’s *d* with pooled standard deviation. The criteria as that defined by Cohen (1988) is being used in which *d* ≥ 0.2 is a small effect, *d* ≥ 0.5 is a medium effect, and *d* ≥ 0.8 is a large effect. The results (see **Table 3**) showed that there were no pre-test group differences in the GraphoLearn tasks. Although effect size was small for letter-sounds (0.30) in favor of the control group, the confidence interval crossed zero. At post-test, group differences in favor of the GraphoLearn group were significant for all GraphoLearn tasks; letter-sounds (*t*(27) = 5.73, *p* = 0.000), rime units (*t*(27) = 2.31, *p* = 0.029), and word recognition (*t*(27) = 2.07, *p* = 0.048). Effect sizes were large for GraphoLearn letter-sounds (2.13) and GraphoLearn rime units (0.85), and medium for GraphoLearn word recognition (0.77), however, only the GraphoLearn letter-sounds had a confidence interval that did not cross zero (1.22, 3.04).

On the paper-pencil tasks, results revealed no significant differences between the groups at neither pre-test nor post-test (see **Table 4**). Effect sizes (*d*) for the group differences at pre-test were very small and supported the *t*-test finding of no significant group differences in BAS II reading (0.13), TOWRE sight words (0.24), TOWRE non-words (0.23), and spelling (0.24). Effect sizes for the paper-pencil tasks at post-test were also very small and again supported the *t*-test finding of no significant group differences in BAS II reading (0.03), TOWRE sight words (0.08), TOWRE non-words (0.09), and spelling (0.05). Confidence intervals for all paper-pencil measures crossed zero at both pre-test and post-test.

### Group Comparisons of Development From Pre-test to Post-test

Repeated measures ANOVA was used to compare the effects of time (change from pre-test to post-test), group (GraphoLearn versus control), and time^∗^group interaction on the scores (group differences in change).

**FIGURE 3:**
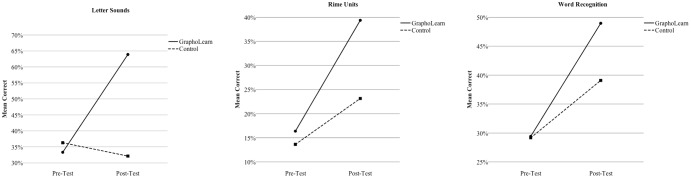
Group Comparisons of Development from Pre-test to Post-test on GraphoLearn Tasks.

**FIGURE 4:**
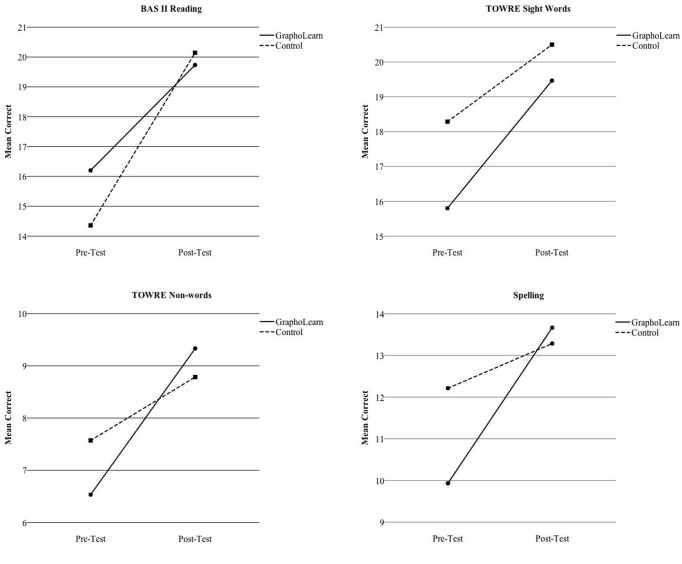
Group Comparisons of Development from Pre-Test to Post-Test on Paper-Pencil Tasks.

For the GraphoLearn tasks (letter-sounds, rime units, and word recognition), there was a significant main effect of time on all three tasks (See **Table 3**), with both groups showing improvement from pre- to post-test (see **Figure 3**). For the letter-sounds task, there was a significant main effect for group, as well as a significant interaction effect for time^∗^group, with the GraphoLearn group showing significantly higher scores and faster development than the control group. For the rime unit task, there were no significant main effects for group or interaction effects for group^∗^time. However, the *p*-values for both the main effect and interaction effect were close to the 0.05 significance level (*p* = 0.09). Finally, for the word recognition task there were no significant group effects or interaction effects for group^∗^time. For the paper-pencil tasks (BAS II reading, TOWRE sight words, TOWRE non-words, and spelling), there was a main effect for time on all measures (see **Table 4**), with both groups showing improvements from pre to post-test (see **Figure 4**). There were, however, no significant effects of group, nor were there significant time^∗^group interactions for the paper-pencil assessments.

### Group Comparisons of Gain Scores

Finally, groups were compared using gains scores. Gain scores were calculated by subtracting the pre-test score from the post-test score for each individual. Means and standard deviations of the gain scores for both groups are given in **Table 5**, along with group comparisons, effect size (Cohen’s *d*), and confidence intervals for the effect sizes for GraphoLearn versus control. The standard errors of the effect sizes are given in parentheses.

**Table 5:** Means and effect sizes of group differences in gains.

Measure	GraphoLearn *M* (*SD*)	Control *M* (*SD*)	*t*	Effect Size *d* (*SE*)	Confidence interval (95%)	
					Lower	Upper
*n*	15	14				
GL letter-sounds	30.57% (15.78)	−4.17% (11.67)	*t*(27) = 6.70^∗∗∗^	2.49 (0.35)	1.52	3.46
GL rime units	22.98% (19.82)	9.51% (21.16)	*t*(27) = 1.77	0.66 (0.24)	−0.09	1.41
GL word recognition	19.53% (13.01)	9.91% (18.35)	*t*(27) = 1.64	0.61 (0.18)	−0.17	1.35
BAS II reading	3.53 (7.03)	3.43 (4.09)	*t*(27) = 0.05	0.02 (0.44)	−0.71	0.75
TOWRE sight words	3.67 (1.25)	2.21 (4.71)	*t*(27) = 0.82	0.30 (0.27)	−0.43	1.04
TOWRE non-words	2.80 (4.04)	0.64 (3.46)	*t*(27) = 1.54	0.57 (0.30)	−0.17	1.32
Spelling	3.73 (3.86)	1.07 (3.61)	*t*(27) = 1.92	0.71 (0.28)	−0.04	1.46

*^∗∗∗^p ≤ 0.001.*

In regards to the GraphoLearn tasks, there was a very large effect on the letter-sound task (2.49) and the confidence interval did not cross zero (1.52, 3.47), allowing us to conclude of a significant difference in favor of the GraphoLearn group. There were medium effects for the rime units (0.64) and word recognition (0.52) tasks, however, confidence intervals on these measures crossed zero. In regards to the paper-pencil tasks, GraphoLearn group versus control group comparison had medium effect sizes on TOWRE non-word reading (0.62) and spelling (0.74). Effect size was small for TOWRE sight word reading (0.31) and almost zero for BAS II single-word reading. Confidence intervals for all paper-pencil measures crossed zero (see **Table 5**).

## Discussion

The present study examined whether GraphoLearn, a computer-assisted reading tool, could effectively support the development of basic English reading skills of struggling readers in India. The participants were Grade 3 slum children in India, who were learning English as a non-native language and who typically had no exposure to English outside of the school environment. Students were divided into either the control or experimental group with the control group playing a simple math game and the experimental group playing GraphoLearn for 20–30 min per day, over a period of 8 weeks. Despite a short play period (∼7.5 h) and limited sample size, participants made significant gains and effect size was promising for at least the letter-sound knowledge, a critical skill for early reading development.

The GraphoLearn intervention group showed the greatest improvements on the letter-sounds task. Group differences were significant, effect size of the gains from pre to post-test was large, and the confidence interval of the effect size did not cross zero, thus allowing us to conclude that there was in fact an effect of the intervention on the difference between the two groups for the letter-sounds knowledge task.

The results show that GraphoLearn can effectively support the development of English letter-sound knowledge in Indian children, despite the fact that participants were non-native speakers and were exposed to the intervention for a limited amount of time. The ability for GraphoLearn to support the development of letter-sound knowledge to this extent is of importance as letter-sound knowledge has been identified as a critical building block in early reading development, even for non-native readers of English (Muter and Diethelm, 2001). There is also evidence in favor of letter-sound knowledge affecting early literacy skills, particularly word reading (Hulme et al., 2012). GraphoLearn can be seen as a beneficial intervention even for bilingual children supporting the previous finding suggesting that bilingual children can benefit just as much as native English speakers when they are provided with literacy interventions that involve explicit emphasis on grapheme-phoneme relationships (Lesaux and Siegel, 2003).

Although the rime unit and word recognition tasks had effect sizes that were medium to large, confidence intervals crossed zero. Due to our small sample size, it is difficult to obtain significant results, and therefore, future studies will need to be done to study the effects of GraphoLearn English with a larger sample. The lack of significant effects may also be partially due to the short playtime. Participants in this study were non-native speakers of English and only had about 7.5 h of play time, as compared to 11 h in the study done by Kyle and colleagues with native speakers of English (Kyle et al., 2013). Due to the structure of the game, only about 60% of participants reached till stream eight, where the explicit practice of all rime units and their accompanying whole words begin. Further studies are required to determine if greater play time will produce significant effects on the GraphoLearn rime units and word recognition tasks.

Paper-pencil measures of reading and spelling were conducted to determine if there was a transfer of skills learned in-game to a non-game assessment. Although effect sizes of the gains were medium for the non-words and spelling tasks, confidence intervals of the effect sizes crossed zero and reflects insignificant group differences. Due to a lack of availiable measures standardized against such populations, we used measures which were designed for native English speaking children. Unfortunately, however, this created a less than ideal testing situation as the tasks were also quite far from what the game explicitly taught. In addition, given the fact that none of the participants had enough time to finish the game, there were many items (e.g., complex GPC’s such as “the rule of *e*”) that participants were not exposed to and therefore, were not able to learn from the game but were required on the paper-pencil measures. Like the in-game assessments, further studies will be required to determine if longer exposure to the game will produce transferable skills. It is also important that future studies use measures which are standardized to such populations.

Overall, the intervention opened the doors for GraphoLearn to be a potential success in the Indian context where the importance of English grows, yet supports for learning the language are lacking for many. We are hopeful that future studies using a larger sample, greater play time, and more effective measures will allow GraphoLearn to be comparable with the few interventions studies that have been done using phonics programs in the Indian setting (e.g., Nag-Arulmani et al., 2003; Dixon et al., 2011), with comparatively less demand of resources. GraphoLearn, as an tool, works by combining successful aspects of previous interventions, while providing individualized learning for students and easy to access data for teachers, factors crucial for implementation and success in a country like India (Central Square Foundation, 2015; Muralidharan et al., 2017). Generalizability of these results will be of question and therefore, it is important that going forward, further testing be done to determine if results improve when the GraphoLearn is used over a longer period of time, with a larger population, and in other parts of India where demands may differ. Nonetheless, this study provides a good first step in looking at how technology, and in particular GraphoLearn, can be used to support the English reading skills of struggling readers in India.

### Limitations

There are a few limitations that must be taken into consideration when evaluating the results of this study. As mentioned, one major limitation was a small sample size. With a sample size of only 30 children, we were limited by the statistical approaches that could be used on the data, and understand that with a bigger sample, we would have had more statistical power. The small sample size also provided us with a limited capacity to control for unobserved variables, therefore, although we had random assignment, the methodological rigor of this random assignment can only be considered as “moderate.” A second limitation was limited intervention time. Although the study was carried out over 8 weeks, the students only played for about 7.5 h. Most inability to play was due to student absenteeism and/or the school being unexpectedly closed. Due to limited play time, no student was able to complete all the streams. Although these factors limit the results of this study, such problems are very real for teachers in India. Therefore, what we see as limited may be what we would actually see if teachers were expected to carry out such and intervention themselves. Third, a methodological limitation that must be considered is the repeated exposure of the GraphoLearn group to the in-game assessments. As previously mentioned, GraphoLearn is built in a way so that students are exposed to an assessment stream after every four practice streams. Thus, students who played GraphoLearn has repeated exposure to the in-game assessments throughout the intervention period, whereas the control group was only exposed to the in-game assessments once at pre-test and once at post-test. This was unavoidable as the in-game measures were necessary to test the skills exactly as taught by the game. Also, using paper-pencil measures which were standardized for native English speakers, made them somewhat difficult for the participants of this study. In the future, this could be avoided by developing experimental measures which are standardized to this particular population. A fourth limitation from the point of view of practical implications was the full-time presence of a researcher during the intervention period. The presence of an adult who was fully focused on the participating children may have increased motivation. The researcher was also constantly supporting students by calling them if they were not in school and making it possible for them to play any time of the school day. In implementation of the game in everyday practices these conditions are not realistic. Similarly, we as researchers had access to a sufficient amount of equipment and resources (i.e., tablets and headphones, a working internet connection) in order for children to be able to play regularly. Going forward it is important that futures studies take into consideration the realities of implementation as to increase chances of sustainability (Central Square Foundation, 2015). Future studies could also study cost-effectiveness of GraphoLearn as an intervention tool in such localities. Finally, based on the current study, we do not know how the effects will be maintained over time. In future studies, it would be important to conduct follow-ups and determine whether or not effects are maintained by students even post-intervention. Going forward, it would also be important to use assessments which are normed for Indian students as to get more accurate results.

### Practical Implications

The current study sheds insight into the ability of computer-assisted reading tools, like GraphoLearn, to support children who struggle to read in India. A logical next step would be to test GraphoLearn English on a larger scale over a longer period. As mentioned previously, the exposure time of students to the game was quite limited due to many uncontrollable factors. Thus, future studies should focus on exposure over a longer duration to determine whether that boosts effects and leads to students being able to transfer the skills they learn in the game to real life situations.

GraphoLearn also opens doors to the ability to provide interventions in children’s mother tongue and other native languages. According to the 2001 census, 41% or more than 422 million individuals in India are Hindi speakers. Despite the large number of speakers, there is still a great need for ed-tech developers to cater to students who are studying in a native language in India (Central Square Foundation, 2015).

By now it has become clear that technology has potential to enhance learning, particularly in developing countries where differentiation is necessary, but difficult for a teacher alone to achieve (Muralidharan et al., 2017). However, there are still critical considerations that must be taken into account prior to implementing technology in schools. According to The World Bank (2018), technology should be used as a complement to teachers rather than a replacement for teachers. A study in India where children were provided technology as a teacher substitute within the school versus a teacher compliment out of school showed that children in the within school group learned significantly less (Linden, 2008). As suggested by Muralidharan et al. (2017), it may be most efficient if technology is used to create what they call a “blended learning” environment in which teachers use the information that they can gather from the technology to guide further instruction. In the current study, GraphoLearn was used as an in-school intervention which was meant to supplement teacher instruction. However, because teachers were not using phonics methods to teach English, there was no teacher involvement and therefore it became an isolated activity that the children performed during the day. In a previous study which looked at the effectiveness of GraphoLearn in Zambia, it was shown that an intervention design in which both students and teachers were trained on and played GraphoLearn lead to the greatest improvements in student learning (Jere-Folotiya et al., 2014). Thus, it must be considered how the technology can be used in greater collaboration with teachers as well. GraphoLearn could provide teachers in India with an alternative to the currently used “rote-memorization” approach, and further increase the use of phonics as a method to teach English literacy in India.

## Author Contributions

PP performed this study as part of her master’s thesis (Patel, 2018). She collected the data and is the main author of the paper. MT supervised PP in her master’s thesis and supported planning of data collection, data analysis, and writing of the paper. MA provided guidance on the writing and proofreading of the paper. UR and HL provided their expertise on the details of the GraphoLearn software, as well as supporting the writing and proofreading of the paper.

## Conflict of Interest Statement

The authors declare that the research was conducted in the absence of any commercial or financial relationships that could be construed as a potential conflict of interest. The reviewer JF declared a shared affiliation, with no collaboration, with one of the authors UR to the handling Editor.

## Footnotes

1 http://info.grapholearn.com/
